# DivStat: A User-Friendly Tool for Single Nucleotide Polymorphism Analysis of Genomic Diversity

**DOI:** 10.1371/journal.pone.0119851

**Published:** 2015-03-10

**Authors:** Inês Soares, Ana Moleirinho, Gonçalo N. P. Oliveira, António Amorim

**Affiliations:** 1 IPATIMUP, Institute of Molecular Pathology and Immunology of the University of Porto, Rua Dr. Roberto Frias s/n, 4200-465, Porto, Portugal; 2 Faculty of Sciences, University of Porto, Rua do Campo Alegre s/n, 4169-007, Porto, Portugal; 3 IFIMUP and IN—Institute of Nanoscience and Nanotechnology, Rua do Campo Alegre, 687, 4169-007, Porto, Portugal; National Institute of Plant Genome Research (NIPGR), INDIA

## Abstract

Recent developments have led to an enormous increase of publicly available large genomic data, including complete genomes. The 1000 Genomes Project was a major contributor, releasing the results of sequencing a large number of individual genomes, and allowing for a myriad of large scale studies on human genetic variation. However, the tools currently available are insufficient when the goal concerns some analyses of data sets encompassing more than hundreds of base pairs and when considering haplotype sequences of single nucleotide polymorphisms (SNPs). Here, we present a new and potent tool to deal with large data sets allowing the computation of a variety of summary statistics of population genetic data, increasing the speed of data analysis.

## Introduction

The most widely-used software packages, such as DnaSP [1] and Arlequin [2] cannot handle the data formats adopted by massive re-sequencing projects. The development of potent tools to analyze the genetic variation of large scale data stored in the variant call format (VCF) developed by the 1000 Genomes Project that has been adopted by other projects, such as UK10K, dbSNP and the NHLBI Exome Project, became imperative [3,4,5]. Recently a program package was designed to provide a number of methods for working with VCF files (VCFtools): validating, merging, comparing and calculate some basic population genetic statistics [6]. It is a Perl based tool that uses the power of Linux/Unix environments through system calls. We have developed a new and robust algorithm, which runs on DivStat software, which uses the power of Linux/Unix, Macintosh and Windows environments, reducing the learning curve for those users less familiar with the shell commands. The program is implemented with a command line shell and also with a user-friendly graphical interface that facilitates algorithm use. This tool can be applied to either polymorphism data or DNA sequences. Moreover, it can compute sequentially a variety of summary statistics of population genetic data over a "sliding window”. After each estimation, the window is slid across the surveyed area and new similar computations can be done. This type of analysis is used in several population genetic studies, allowing for the inspection of variation patterns along genomic fragments. In this paper we describe the DivStat software and demonstrate its usefulness. Furthermore, we also compare it with other tools and illustrate their capabilities. The algorithm details, tutorial, software, clear and explicit examples that users can use to test both versions of DivStat software and also output files showing the results form hypothetic studies are freely available as supplementary material of this manuscript at https://www.mediafire.com/folder/za5wjlcoc5oi1/DivStat and http://www.portugene.com/DivStat.html.

## Material and Methods

We here describe a new tool that allows a simultaneous feed and analysis of large data sets. The developed algorithm was implemented into a program that allows for the computation of different statistics of population genetic data, comprising a number of DNA haplotype sequences encompassing more than a thousand of base pairs. The designed program accepts input files in VCF or fasta format, including both complete DNA sequences or SNP haplotypes. We designed a user-friendly interface in order to facilitate the use by the research community, allowing the upload of a file in the VCF format or a text file with the genetic data in the fasta format. Moreover, a command line version was also developed, allowing the upload of a folder with more than a VCF or text file.

### Algorithm

**Fig 1:**
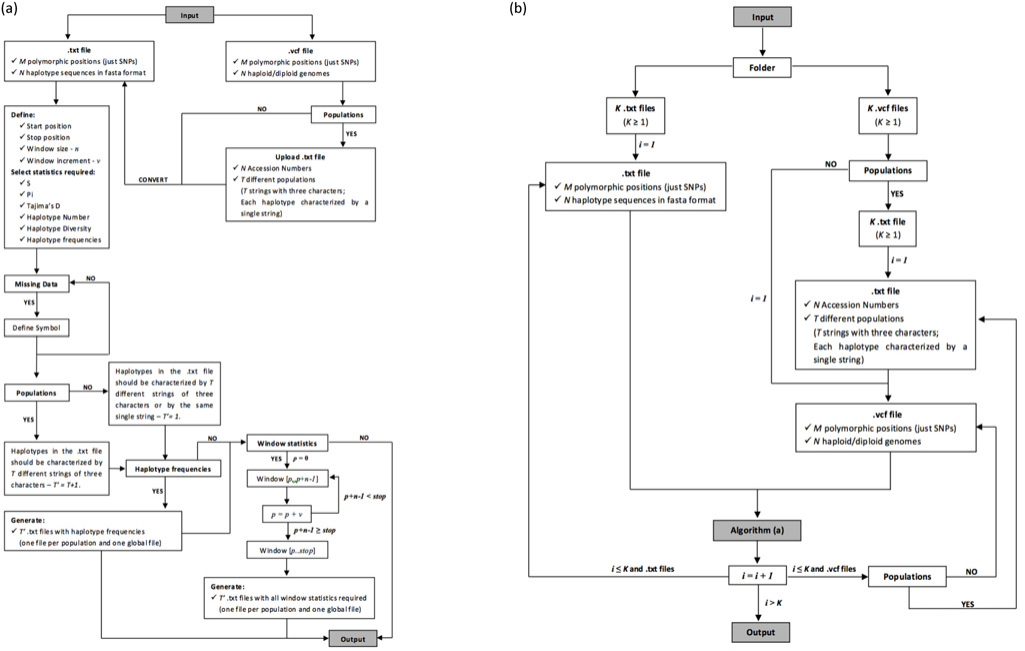
Design of DivStat algorithm: (a) GUI version; (b) cmd version.

An overview of the software, showing the algorithm details is presented in Fig. 1. The modified waterfall model was the process adopted to develop the DivStat approach and the full step-wise procedure is described in S1 Table.

### Program features

The statistics that DivStat calculates can be performed for a single population sample or for multiple population samples. Furthermore, this tool allows for the computation of several statistics within a window with a user definable length. The full list of statistics is givenbelow. First, the users should define a set of parameters, namely, the start and end positions of the segment, the window size and the window increment. When using polymorphism data, the numbering of site positions within the file must be consistent with the numbering used to define the segments. Thus, for instance, defining a window size of *n* and considering *p* as its start position, the program calculates the statistics within the window [*p*.*p*+*n*-*1*]. If the window increment is *v*, it means that the next computations are done after sliding the window of *v* base pairs, i.e., in the window [*p*+*v*.*p*+*v* +*n*-*1*].

The algorithm starts by assigning the digits 1, 2, 3 and 4 to bases A, C, G and T, respectively (similarly to the methodology adopted in [7]), and 5 to missing data, and then each sequence is converted into a vector according this numerical correspondence. Considering a dataset encompassing *N* haplotype sequences, each with *M* sites, a matrix *X* with *M* rows and *N* columns is constructed after the numerical correspondence. Based on *X*, the program allows for the quick computation of six statistics (for details, please see S1 File):

1. *S*: *T*he number of polymorphic sites that are contained within the window.
2. *Haplotype number*: The number of different haplotypes within the window.
3. *Haplotype diversity*: The diversity of each haplotype within the window, which is given by the following as in [8]:
  $$Hd=\frac{N}{N-1}\left(1-\sum_{j}Hf{\left[j\right]}^{2}\right)$$
  where *Hf*[*j*] is the frequency of the *j* ^th^ haplotype in the window.
4. *π*: The nucleotide diversity within the window. This statistic is computed based on the nucleotide diversity of each single polymorphic site *i* in the window, with *k* different alleles, usually represented as π_*i*_ (the formula for π_*i*_ is given in the Supplementary Information). The nucleotide diversity for a window containing *S* polymorphic positions is then computed according with [9]:
  $$\Pi =\sum_{i=1}^{S}\pi_{i}$$
  And, being *n* the size of the window, the nucleotide diversity per base pairs is calculated as:
  π=Π/n
5. *Tajima’s D*: Tajima’s D is a statistic that allows for assessing the evidence or not of selection in the data set. The computation of this statistic is based on the Watterson’s *θ*_*w*_ and *var*(*π-θ*_*w*_) (formulas given in the Supplementary Information), being computed by the following formula as in [9]:
  $$D=\left(\Pi -\theta_{w}\right)/\sqrt{Var\left(\pi -\theta_{w}\right)}$$
  In the case of *var*(*π-*θ_*w*_) is null, the value of *D* cannot be computed (in this case, in the output file produced by DivStat, the corresponding value of Tajima’s D statistic will be represented by “?” symbol).
6. *Haplotype frequencies*: we developed an algorithm that starts by determining all different haplotypes in the dataset and then computes the haplotype frequencies. This calculation is independent of the window size and the increment defined.

### Data

Our approach aims to analyze small and large data sets of DNA sequences and haplotypes of polymorphic sites in the text format as well as in the VCF format, easily and quickly converted into the accepted text format before computing the population statistics. In fact, the software was trained, tested and validated by a large collection of data sets that were obtained by combinations of a small or great amount of sequences or haplotypes with a few or more than a thousand of base pairs or polymorphisms.

### Software

The algorithm was written in Python, version 2.7.6, and tested on Macbook pro, Mac OS X 10.9 system with an Intel(R) Core i7 CPU @ 2.3 GHz and 8b RAM, Linux—Ubuntu 12.10 and Windows 7–32bit system with an Intel(R) Core(TM)2 Duo CPu, T9300 @ 2.50 GHz and 4Gb of RAM and 64bit system with an Intel(R) Pentium (R) Dual CPU, T3400 @ 2.16 GHz and 4Gb of Ram. The interface, created using a tool prepared to interact with the Python programming language—VisualWx, was designed to run in Macintosh, Unix and Windows 7 systems.

## Results

The developed algorithm starts by a pre-analysis of the inputted data in order to verify whether the information was correctly inserted. If some inconsistency is detected, namely files and/or fields outside the standard accepted format, the software aborts and alerts the user to check the data. The program identifies the field that is not filled according to the DivStat standard rules, facilitating the correction of the data and the restarting of the analysis. When the data have the correct format and the fields are filled correctly, DivStat efficiently computes the desired statistics. Examples of the specific file formats accepted in DivStat are available with the setup of the software. Furthermore, a tutorial for the software was added and can be accessed directly on the software or be consulted as supplementary information.

The software was satisfactorily tested with the genomic data of several hundreds of haplotype sequences. To demonstrate the usage and efficacy of DivStat, we provide two examples (S1 Data). The developed algorithm was tested in a training set of haplotype sequences from 1,092 individuals belonging to 14 populations. The data set contains 1707 SNPs spread along 100000 base pairs [10]. It was also applied to a larger data set of 40 DNA haplotype sequences from 20 individuals belonging to the same population, containing 77754 SNPs spread along 3800000 base pairs.

### Program performance

The developed algorithm for statistics estimation proved to be computationally very fast, allowing for the simultaneous upload and analysis of large datasets. When performing the analysis of the first training set per populations, considering a window size of 2000 base pairs and an increment of 150 base pairs each time, the computation of all statistics was accomplished in 26 minutes and 25 seconds; when considering all haplotypes as a single group, and assuming the same parameters, the calculation of all statistics was carried out in 13 minutes and 23 seconds. Using the second training set, a window size of 10000 base pairs and an increment of 9000 base pairs was defined and, the computation of all statistics was accomplished in 2 hours, 43 minutes and 44 seconds. (Just the running times for the windows–32bits system were considered here. For other tested platforms, the algorithm showed a similarly high performance, exhibiting running times of the same order of magnitude.) Irrespectively of the type of the data set used, the developed algorithm showed a good performance, being fast and presenting the expected results.

### Performance Comparison between DivStat and available tools

The features and performance of DivStat software was compared to other available tools with similar purposes, namely, SLIDER and VCFtools (http://genapps.uchicago.edu/slider/index.html and [6]). A thoroughly and fair comparison of our and these previous tools is unfeasible, since they handle different types and amounts of data. Therefore, we were only able to perform an in silico comparison, based on some features that are shared between Divstat and the other two tools. The capabilities and characteristics of each tool are illustrated in S2 Table and are described in the following subsections. The results obtained from comparison analyses are available in the (S2 Data).

#### DivStat VS VCFtools

VCFtools only accepts polymorphism data in the VCF format, contrarily to DivStat that can deal with both polymorphism data and DNA sequences, in the VCF or fasta format. The fasta format supported by Divstat is an asset to our approach, since it considerably compresses the size of the data set. For instance, considering the same polymorphism data, summarized in S2 Table, in the VCF format the file size is ~2.4Mb and in the fasta format supported by DivStat it is just ~200Kb. In terms of running time, considering the data set summarized in S2 Table, DivStat takes 27 seconds to generate the output file whereas VCFtools runs each command in 1 second. VCFtools is faster than DivStat, nevertheless, it requires the instruction of a command one by one to compute the desired statistics, whereas DivStat can compute all required statistics at the same time. Furthermore, only 3 statistics, S, Pi and Tajima’s D are common to both VCFtools and Divstat, but we can only compare the results obtained over a sliding window, in which the window incrementally advances across the surveyed region, for S and Pi statistics. For tajima´s D computation, VCFtools does not allow the window to advance by adding an increment but only in contiguous non-overlapping windows. Additionally, DivStat is compatible with three operating systems, namely, Macintosh, Unix and Windows, while VCFtools only runs on the Linux/Unix platform.

#### DivStat VS SLIDER

SLIDER has a very strict maximum dataset size (1MB) and only accepts polymorphism data in file formats that increase considerably the size of the data set. For instance, considering the same polymorphism data, summarized in S2 Table, in the format accepted by SLIDER, the size of the file is ~1Mb and in the fasta format supported by DivStat it is just ~340Kb. Both tools can be configured to compute the statistics over a sliding window, but they could never be compared under the same computational requirements, since SLIDER is a web-based tool that runs on an online server. DivStat is compatible with three environments (Macintosh, Unix and Windows), while SLIDER just requires an internet connection. Considering the data set summarized in S2 Table, to generate the same output data, DivStat only takes 2 minutes and 52 seconds whereas SLIDER needs 9 minutes and 28 seconds. It is worth noting that the running time of SLIDER could vary depending on the internet connection speed available.

## Discussion

Most of the existing programs for computing a variety of summary statistics of population genetic data use DNA sequence. The software here described represents a new tool to efficiently use, not only DNA sequences but also polymorphism data, like those recently released in the VCF format. We have compared the improvement of our approach over other available methods having similar purposes, namely VCFtools and SLIDER. Unfortunately, from a practical perspective, it is quite difficult to perform a comparison between them because they handle different types and amounts of data. Nevertheless, we performed the reasonable comparisons taking into account some shared features. VCFtools only accepts polymorphism data in the VCF format, whereas DivStat can deal with both, polymorphism data and DNA sequences in VCF or fasta format. For the inspection of variation patterns along genomic fragments, DivStat can be configured, so that its window size is defined either in terms of a fixed number of base pairs or a fixed number of segregating sites, whereas VCFtools only allows the first option. In fact, when doing a whole-genome analysis, it is suitable to calculate some statistics, such as nucleotide diversity or Tajima's D, on a fixed number of segregating sites, because it would be hard to compare values across the genome in fixed bp windows, since some windows will have fewer sites while other will be densely populated. However, when genetic variation is used to investigate particular genomic regions, it is more accurate to compute those statistics per base pair, since the existence of big gaps with no diversity is a common feature of the eukaryotic genome landscape. Moreover, VCFtools only runs on a Linux/Unix environment whereas DivStat is compatible with three operating systems: Macintosh, Unix and Windows, and is implemented with a command line shell and also with a user-friendly graphical interface which does not require computational expertise. Concerning SLIDER (http://genapps.uchicago.edu/slider/index.html), it also computes a variety of summary statistics of population genetic data over a "sliding window”, which size can be defined by number of base pairs and by number of polymorphic sites. Nonetheless, contrarily to DivStat, aiming to apply the second option in SLIDER, the user cannot input DNA sequences but only polymorphism data. Additionally, SLIDER does not support VCF format, so it is necessary first to convert the data into a Fasta format. Furthermore, it has a very strict dataset size (1MB) and is a web-based application that runs on an online server, requiring thus an internet connection, so the internet speed and also the availability of the server are the main factors influencing the running time of SLIDER. Our approach allows for the simultaneous feed and analyses of large data sets, and also provide a tool that allows users to upload more than a data file simultaneously, making the analysis quicker and more effective. Since in terms of accuracy the three tools are identical, choosing between them depends primarily on the type and size of data in hand.

## Supporting Information

S1 Table DivStat algorithm: (a) GUI algorithm; (b) cmd algorithm. (DOCX)

S2 Table Summary comparison between (a) DivStat and VCFtools and (b) DivStat and SLIDER performances. (DOCX)

S1 File Supplementary Information. (DOC)

S2 File Read Me. (TXT)

S3 File Tutorial. (PDF)

S1 Data Test Data. (RAR)

S2 Data Performance Comparisons. (RAR)

S1 DivStat DivStat Windows version. (RAR)

S2 DivStat DivStat Linux version. (RAR)

S3 DivStat DivStat MacOS-GUI version. (DMG)

S4 DivStat DivStat MacOS-cmd version. (DMG)
