# Supplementary material for: DivStat: A User-Friendly Tool for Single Nucleotide Polymorphism Analysis of Genomic Diversity
Source: PLoS One. 2015 Mar 10;10(3):e0119851. doi: 10.1371/journal.pone.0119851 (PMC4355611; doi:10.1371/journal.pone.0119851)
Supplement: S1 Table — (DOCX) [file pone.0119851.s001.docx]

**S1 Table.** DivStat algorithm: (a) GUI algorithm; (b) cmd algorithm

| (a) GUI algorithm |
| --- |
| INPUTS  file: .txt or .vcf file storing the sequence data (N haplotype sequences (HapSeq), each with M sites)  s: start position  e: stop position  n: window size (defined by number of base pairs or number of segregating sites )  v: window increment  MD: missing data symbol (in case of data with missing data)  Stat: set storing the required statistics:  S: number of polymorphic sites  Hn:haplotype number  Hd: haplotype diversity  π: Pi  D: Tajima’s D  hf: haplotype frequencies  Pop: population requirements:  Pop ← YES: if the computation should be performed per populations ( T populations)  file_pop: .txt file storing the population information in case of .vcf file (in case of .txt file, each haplotype sequence should be characterized by a string of three characters).  Pop ← NO: if the computation should not be performed per populations  1: if file is .vcf:  2: file ← converted into .txt format  3: X ← matrix(M,N)  4: for i in range(1,N):  5: for j in range(1,M):  6: if HapSeq[i,j] = A:  7: X[i,j] ← 1  8: if HapSeq[i,j] = C:  9: X[i,j] ← 2  10: if HapSeq[i,j] = G:  11: X[i,j] ← 3  12: if HapSeq[i,j] = T:  13: X[i,j] ← 4  14: if HapSeq[i,j] = MD:  15: X[i,j] ← 5  16: if Pop = YES:  17: T’ ← T+1 (compute the statistics for each population separately and also for the global set of haplotype sequences)  18: if Pop = NO:  19: T’ ← 1 (compute the statistics just for the global set of haplotype sequences)  20: for p in range (1:T’):  21: X’ ← matrix(M,N’) (a sub-matrix of X that just considers the haplotype sequences in population p; N’ represents the number of haplotype sequences in population p)  22: if hf in Stat: (Computes the haplotype frequencies (hf) according to equation (6) in the Supplementary Information.)  23: H ← # different columns of X’  24: for i in range(1:H):  25: h[i] ← # columns of X’ equal to i^th^ different column  26: $hf\left[i\right]\leftarrow h\left[i\right]/N'$  27: if ((S or Hn or Hd or π or D) in Stat):  28: p ← s  29: while (p+n–1 ≤ e):  30: Y ← X’[p…p+n-1,N’]  31: if ((S or π or D) in Stat): (Computes S according to (1) in the Supplementary Information.)  32: S ← # non-conserved rows of Y  33: if ((Hn or Hd) in Stat): (Computes Hn according to (2) in the Supplementary Information.)  34: Hn ← # different columns of Y  35: if (Hd in Stat): (Computes Hd according to equations (3) in the Supplementary Information.)  36: for j in range(1:Hn):  37: H[j] ← # columns of Y equal to j^th^ haplotype.  38: $Hf\left[j\right]\leftarrow H\left[j\right]/N'$  39: $Hd\leftarrow\frac{N'}{N'-1}\left(1-\sum_{j} Hf\left[j\right]^{2}\right)$  40: if (π or D) in Stat): (Computes π according to equations (4) in the Supplementary Information.)  41: for i in range(1:S):  42: k ← # different entries in the non-conserved row i of Y.  43: for q in range(1:k):  44: x_iq_ ← # columns of Y with an entry equal to the q^th^ different entry at row i  45: $\pi_{i}\leftarrow1-\left(\sum_{q=1}^{k} \left(x_{iq}^{2}-x_{iq}\right)/\left(\left(\sum_{q=1}^{k} x_{iq}\right)^{2}-\sum_{q=1}^{k} x_{iq}\right)\right)$  46: $\Pi\leftarrow\sum_{i=1}^{S} \pi_{i}$  47: $\pi\leftarrow\Pi/n$  48: if D: (Computes D according to equations (5) in the Supplementary Information.)  49: $a_{1}\leftarrow\sum_{j=1}^{N-1} 1/j$  50: $a_{2}\leftarrow\sum_{j=1}^{N-1} 1/j^{2}$  51: $b_{1}\leftarrow\left(N'+1\right)/\left(3\left(N'-1\right)\right)$  52: $b_{2}\leftarrow\left(2\left(N'^{2}+N'+3\right)\right)/\left(9N'\left(N'-1\right)\right)$  53: $c_{1}\leftarrow b_{1}-\left(1/a_{1}\right)$  54: $c_{2}\leftarrow b_{2}-\left(\left(N'+2\right)/\left(a_{1}N'\right)\right)+a_{2}/a_{1}^{2}$  55: $e_{1}\leftarrow c_{1}/a_{1}$  56: $e_{2}\leftarrow c_{2}/\left(a_{1}^{2}+a_{2}\right)$  57: $\theta_{w}\leftarrow S/a_{1}$  58: $Var\left(\pi-\theta_{w}\right)\leftarrow e_{1}S+e_{2}S\left(S-1\right)$  59: $D\leftarrow\left(\Pi-\theta_{w}\right)/\sqrt{Var\left(\pi-\theta_{w}\right)}$  60: p ← p+v  61: if (p+n–1>e and p<e):  62: Y ← X’[p…e,N’]  63: Repeat lines 31 – 59  OUTPUT  .txt file (s) storing the required statistics |

| (b) cmd algorithm |
| --- |
| INPUTS  folder: folder with K≥1 .txt or .vcf files, which stores the sequence data (each file f ($f\in\left\{1,2,\ldots,K\right\}$) contains N haplotype sequences (HapSeq), each with M sites)  s_1_…s_K_: start position in each file f  e_1_…e_K_: stop position in each file f  n: window size (defined by number of base pairs or number of segregating sites )  v: window increment  MD: missing data symbol (in case of data with missing data)  Stat: set storing the required statistics:  S: number of polymorphic sites  Hn: Haplotype number  Hd: Haplotype diversity  π: Pi  D: Tajima’s D  hf: haplotype frequencies  Pop: population requirements:  Pop ← YES: if the computation should be performed per populations  file_pop_1_… file_pop_K_: .txt files storing the population information of each file f, in case of .vcf file (in case of .txt file, each haplotype sequence should be characterized by a string of three characters). (T populations in the file f)  Pop ← NO: if the computation should not be performed per populations  1:for f in folder:  2: file ← f  3: s ← s_f_  4: e ← e_f_  5: file_pop ← file_pop_f_  6: follow the lines 1 – 63 of GUI algorithm (a)  OUTPUT  .txt files storing the required statistics for the K inputted files. |
