# Supplementary material for: DivStat: A User-Friendly Tool for Single Nucleotide Polymorphism Analysis of Genomic Diversity
Source: PLoS One. 2015 Mar 10;10(3):e0119851. doi: 10.1371/journal.pone.0119851 (PMC4355611; doi:10.1371/journal.pone.0119851)
Supplement: S2 Table — (DOCX) [file pone.0119851.s002.docx]

**S2 Table.** Summary comparison between (a) DivStat and VCFtools and (b) DivStat and SLIDER performances.

|  | | Softwares | | | | | | | | | | | | | | | | | |
| --- | --- | --- | --- | --- | --- | --- | --- | --- | --- | --- | --- | --- | --- | --- | --- | --- | --- | --- | --- |
|  | (a) | | | | | |  | | | | | (b) | | | | | | | |
| Features | | | **DivStat** | **VCFtools** | | | | |  | | | | **DivStat** | | **SLIDER** | | | | |
| Data Size | | 208Kb | | | 2.405Mb | | | | |  | | | | 342Kb | | 999Kb | | |  |
| Running Time | | 27sec | | | 1sec/step* | | | | |  | | | | 2min52sec | | 9min28sec | | |  |
| Maximum Data Size | | - | | | - | | | | |  | | | | - | | 1Mb | | |  |
| Allowed Statistics:  S  Pi  Tajima’s D  Haplotype Number  Haplotye Diversity | |  | | | |  | |  | | |  | | | | | |  | | |
|  |  | x | | | | x | |  | | | x | | | | | | | x | |
|  |  | x | | | | x | |  | | | x | | | | | | | x | |
|  |  | x | | | | x | |  | | | x | | | | | | | x | |
|  |  | x | | | |  | |  | | | x | | | | | | | x | |
|  |  | x | | | |  | |  | | | x | | | | | | | x | |
| Compatible Operative Systems:  Windows  Linux  Max OS | |  | | | |  | |  | | |  | | | | | | |  | |
|  |  | x | | | |  | |  | | | x | | | | | | | x^+^ | |
|  |  | x | | | | x | |  | | | x | | | | | | | x^+^ | |
|  |  | x | | | |  | |  | | | x | | | | | | | x^+^ | |

* The software computes only one statistic each time, and each step runs in 1sec.

^+^ The software runs in an online server.
