# Supplementary material for: DivStat: A User-Friendly Tool for Single Nucleotide Polymorphism Analysis of Genomic Diversity
Source: PLoS One. 2015 Mar 10;10(3):e0119851. doi: 10.1371/journal.pone.0119851 (PMC4355611; doi:10.1371/journal.pone.0119851)
Supplement: S1 File — (DOC) [file pone.0119851.s003.doc]

**Supplementary Information:**

# methods

## Program features

The statistics that DivStat calculates can be performed for a single population sample or for multiple population samples. Furthermore, this tool allows for the computation of several statistics within a window with a definable length. The full list of statistics is given below. First, the users should define a set of parameters, namely, the start and end positions of the segment, the window size and the window increment. When using polymorphism data, the numbering of site positions within the file must be consistent with the numbering used to define the segments. Thus, for instance, defining a window size of *n* base pairs and considering *p* as its start position, the program calculates the statistics within the window [*p*..*p*+*n*-*1*]. If the window increment is *v*, it means that the next computations are done after sliding the window of *v* base pairs, ie, in the window [*p*+*v*..*p*+*v* +*n*-*1*].

The algorithm starts by assigning the digits 1, 2, 3 and 4 to bases A, C, G and T, respectively (similarly to the methodology adopted in [1]), and 5 to missing data symbol (in the case it happens), and then each sequence is converted into a vector according this numerical correspondence. Considering a dataset encompassing *N* haplotype sequences, each with *M* sites, a matrix *X* with *M* rows and *N* columns is constructed after the numerical correspondence. Based on *X*, the program allows for the quick computation of six statistics. Considering, for instance, the sub-matrix *Y* of *X* in the window [*p*..*p*+*n*-*1*]:

with $a_{ij}\in\left\{1,2,3,4\right\}$, the different statistics are determined as explained below:

*(1) S*: *S* is the number of polymorphic sites that are contained within the window. The computation of *S* is resumed to the computation of non-conserved rows of *Y* (which corresponds to the rows of *X* falling down within the window [*p*..*p*+*n*-*1*] and having more than one entry).

*(2) Haplotype number*: Haplotype number is the number of different haplotypes within the window. The algorithm developed computes this statistic by determining the number of different columns of *Y*.

*(3) Haplotype diversity*: Haplotype diversity is the diversity of each haplotype within the window. Considering the different haplotypes computed in the previous item, the haplotype frequency is given by the following:

$$Hf\left[j\right]=H\left[j\right]/N$$

where *H*[*j*] and *N* are the number of occurrences of the *j*^th^ haplotype in the window (ie, the number of columns of *Y* equals to haplotype *j*) and the total number of sequences in the data set, respectively. After this, the haplotype diversity is computed as in [2]:

$$Hd=\frac{N}{N-1}\left(1-\sum_{j} Hf\left[j\right]^{2}\right)$$

*(4) π*: π is the nucleotide diversity within the window. This statistic is computed based on the nucleotide diversity of each single polymorphic site *i* in the window, with *k* different alleles, usually represented as π*_i_* and given by the following:

$$\pi_{i}=1-\left(\sum_{q=1}^{k} \left(x_{iq}^{2}-x_{iq}\right)/\left(\left(\sum_{q=1}^{k} x_{iq}\right)^{2}-\sum_{q=1}^{k} x_{iq}\right)\right)$$

where, *x_iq_* is the number of haplotypes within the window with the allele *a_q_* at position *i*, ie, the number of columns of *Y* with *a_qj_*. The nucleotide diversity for a window containing *S* polymorphic positions is computed according with [3]:

$$\Pi=\sum_{i=1}^{S} \pi_{i}$$

And, being *n* the size of the window, the nucleotide diversity per base pairs is calculated as the following:

$$\pi=\Pi/n$$

*(5) Tajima’s D*: Tajima’s D is a statistic that allows for assessing the evidence or not of selection in the data set. The computation of this statistic is based on the Watterson’s and , according to [3], which are given by:

$\theta_{w}=S/a_{1}$ and $Var\left(\pi-\theta_{w}\right)=e_{1}S+e_{2}S\left(S-1\right)$

where, $Var\left(x\right)$ means the variation of *x*, $e_{1}=c_{1}/a_{1}$, $c_{1}=b_{1}-\left(1/a_{1}\right)$, $b_{1}=\left(N+1\right)/\left(3\left(N-1\right)\right)$, $a_{1}=\sum_{j=1}^{N-1} 1/j$, $a_{2}=\sum_{j=1}^{N-1} 1/j^{2}$, $e_{2}=c_{2}/\left(a_{1}^{2}+a_{2}\right)$, $c_{2}=b_{2}-\left(\left(N+2\right)/\left(a_{1}N\right)\right)+a_{2}/a_{1}^{2}$ and $b_{2}=\left(2\left(N^{2}+N+3\right)\right)/\left(9N\left(N-1\right)\right)$.

The Tajima’s D statistic is then computed by the following:

$$D=\left(\Pi-\theta_{w}\right)/\sqrt{Var\left(\pi-\theta_{w}\right)}$$

*(6) Haplotype Frequencies:* We developed an algorithm that starts by determining all different haplotypes in the dataset and then computes the haplotype frequencies according to:

$$hf\left[i\right]=h\left[i\right]/N$$

for the *i*^th^ haplotype, being *h*[*i*] and *N* the number of occurrences of haplotype *i* and the total number of haplotype sequences in the dataset, respectively. This calculation is independent of the window size and the increment defined.

**REFERENCES**

1. Soares I, Amorim A, Goios A (2011) A new algorithm for mtDNA sequence clustering. Forensic Science International: Genetics Supplement Series 3 (1), e315-e316.
2. Nei M and Tajima F (1981) DNA polymorphism detectable by restriction endonucleases. Genetics 97:145-163.
3. Tajima F (1989) Statistical method for testing the neutral mutation hypothesis by DNA polymorphism. Genetics 123 (3): 585–95.
