# Supplementary material for: DivStat: A User-Friendly Tool for Single Nucleotide Polymorphism Analysis of Genomic Diversity
Source: PLoS One. 2015 Mar 10;10(3):e0119851. doi: 10.1371/journal.pone.0119851 (PMC4355611; doi:10.1371/journal.pone.0119851)
Supplement: S3 File — (PDF) [file pone.0119851.s005.pdf]

Tutorial

DivStat is a new software developed in order to help users to analyze great sets of population genetic data stored in VCF (variant call format) files. It allows the computation of six statistics, namely, the number of polymorphic sites – S, Haplotype Number, Haplotype diversity, π, Tajima's D, and haplotype frequencies. They can be computed for the complete DNA fragment or over a "sliding window". First, the users should define a set of parameters, namely, the start and end positions of the segment, the window size (in base pairs or segregating sites) and the window increment in base pairs. When using polymorphism data, the numbering of site positions within the inputted file must be consistent with the numbering used to define the segments.

A user-friendly interface was developed in order to facilitate the use by the research community. The graphical interface allows the upload of a VCF file or a text file with the genetic data in the fasta format. Moreover, a command line version was developed, allowing the upload of a folder with more than a VCF or text file.

1. *Graphical User Interface version – GUI:*

   When the user opens the DivStat software GUI version, the following window appears on the screen:

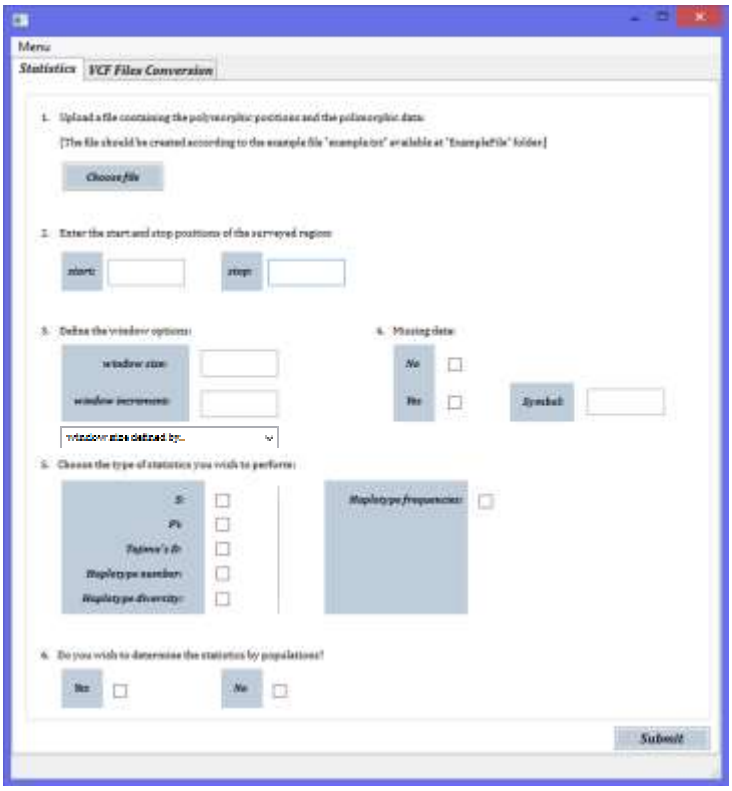

Figure 1. GUI of DivStat software.

First (1st point), the user should upload a file containing the polymorphism data, which could be a VCF or a text file containing the SNPs and the corresponding position number in the complete genome.

1.1. *Uploading a text file*

If the user uploads a text file containing the SNPs and the corresponding position number in the complete genome, it should be similar to the following examples:

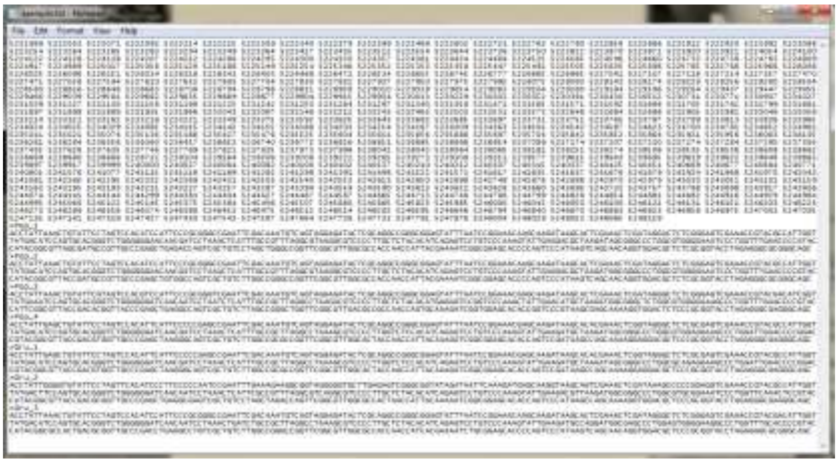

Figure 2. Example of an accepted input text file in the fasta format.

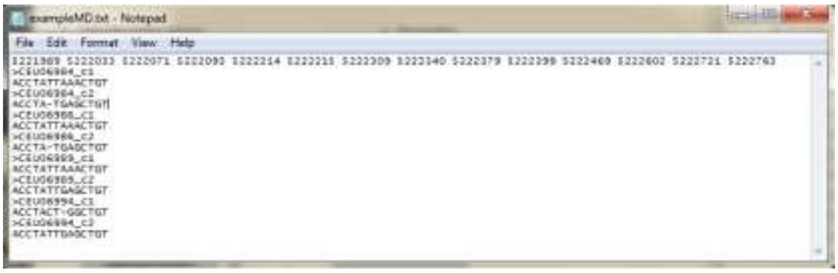

Figure 3. Example of an accepted input text file, in the fasta format, with missing data (represented by symbol “-”).

Note that, the position numbers should be written at the first line of the document, the following ones corresponding to the SNP sequences.

On the 2nd and 3rd points, the user should define a set of parameters, namely, the start and end positions of the haplotype sequences, the window size, defined by number of base pairs or segregating sites, and the window increment. Defining, for example, a window size of *n* base pairs and considering *p* as its start position, the program computes the chosen statistics within the window [*p*..*p*+*n*-*1*], working just with the SNP positions that fall within this interval. If the window increment is *v*, it means that the next computations are computed after sliding the window of *v* base pairs, i.e., in the window [*p*+*v*..*p*+*v* +*n*-*1*].

On the 4th point, the user should indicate whether the data has or not missing data. In the affirmative case, the user should indicate the symbol used.

Finally (5th point), the user should select or deselect the statistics to be performed. Note that, the haplotype frequency computation does not take into consideration the window parameters defined.

The calculations can be done per populations or globally (6th point). The global estimation is computed in both cases, but population specific outputs are only obtained if indicated in this point. To obtain results by population, the first three characters of each line started by “>” should identify the population. For example, on the text file of Figure 2. there are two different populations, “Pop” and “Gru”; on the text file of Figure 3., all eight sequences belongs to the same population, “CEU”.

On the following images, it is possible to see two different examples of fields filling:

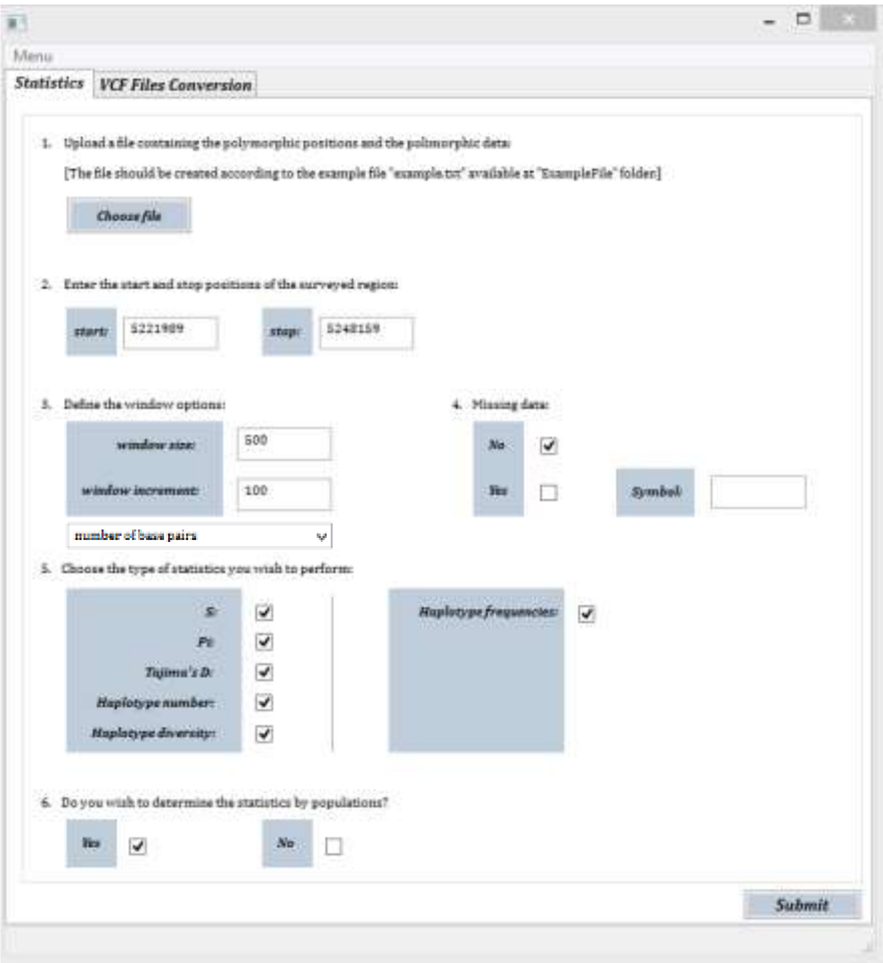

Figure 4. Example of possible preferences using the file of Figure 2.

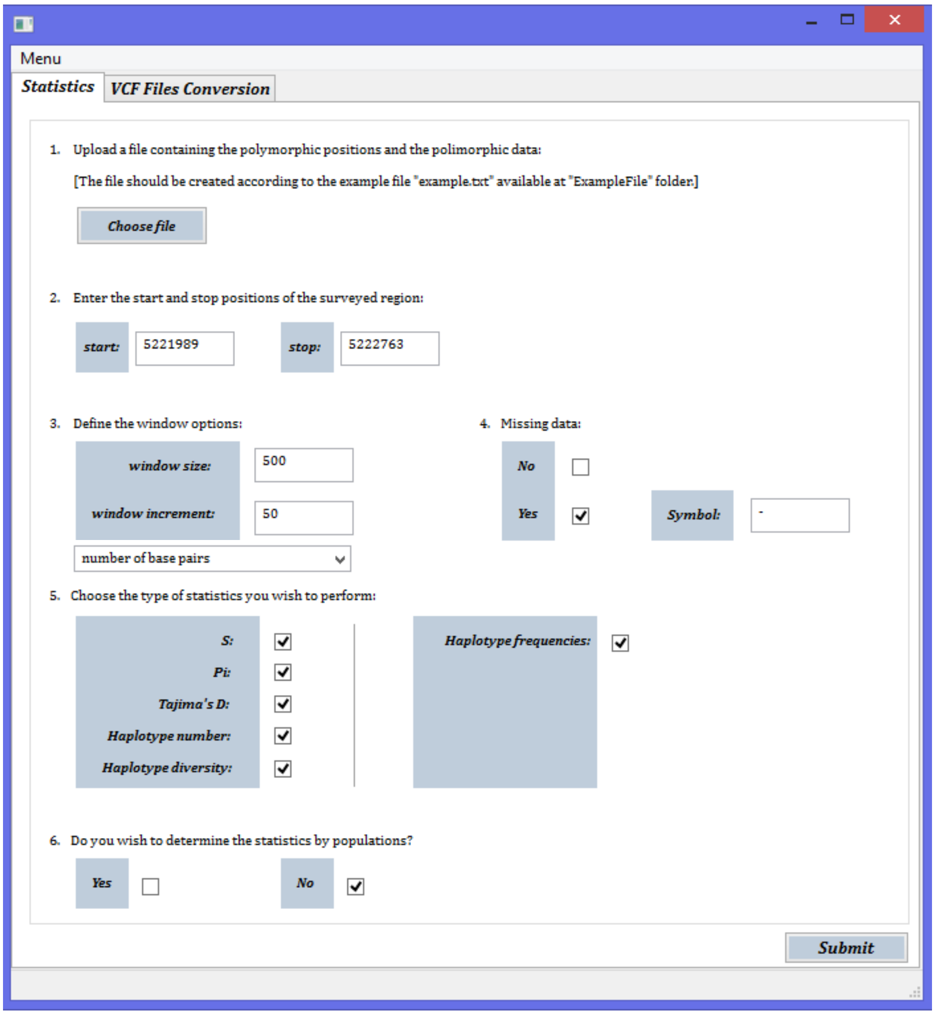

Figure 5. Example of possible preferences using the file of Figure 3.

On the first case (Figures 2. and 4.), the file has not missing data and the required statistics are calculated per population. On the second one (Figures 3. and 5.), the file has missing data, which is identified by symbol "-", and the required statistics are not computed per population. In both cases, the window is defined by number of base pairs.

The haplotype frequencies will be saved on independent files, while all window statistics will be saved on the same file. Furthermore, the output comprises a file per population and a global file (comprising the statistics computed for all sequences considered as a global group). Examples of the output can be seen on the following images:

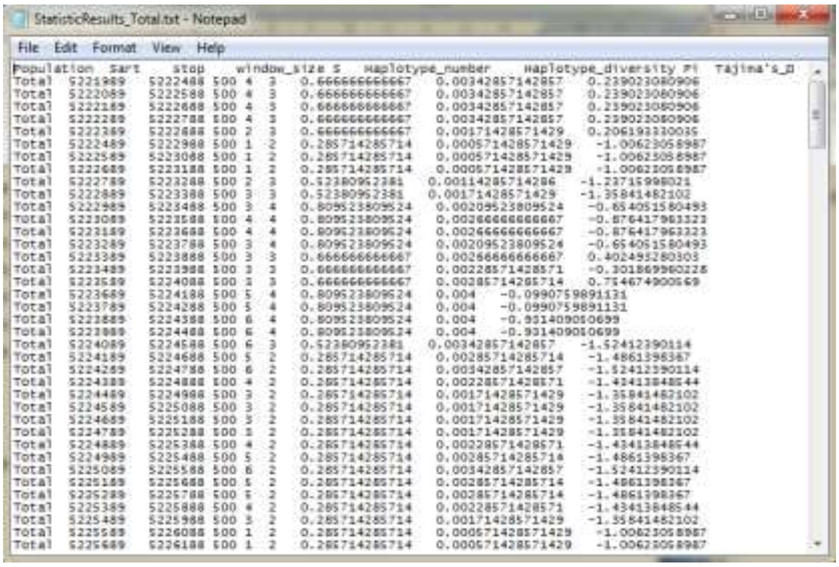

Figure 6. Example of an output (window statistic computation – S, Haplotype Number, Haplotype diversity; π and Tajima's D – computed globally) using the file of Figure 2. and fields filed according Figure 4.

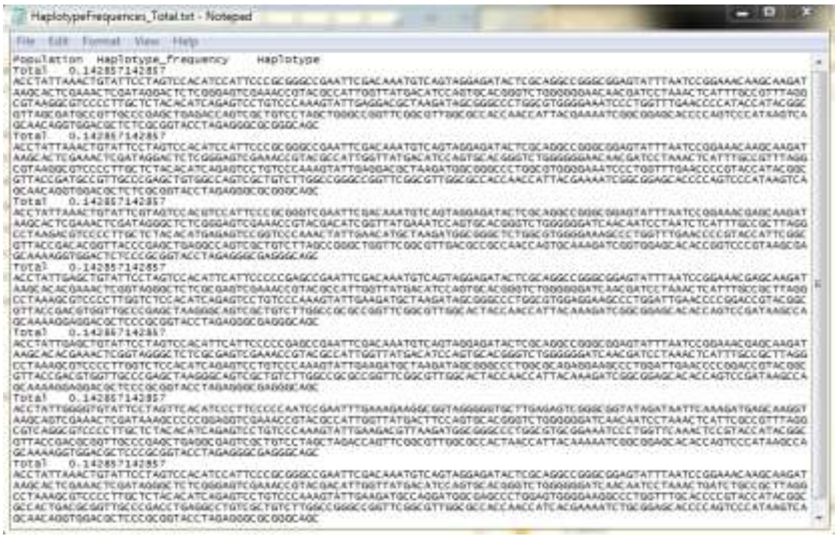

Figure 7. Example of an output for haplotype frequencies using the file of Figure 2. and fields filed according Figure 4.

The shown example files correspond to statistics computed globally. The statistics calculated for specific populations produce similar output files.

1.2. *Uploading a VCF file*

If the user uploads a VCF file, the following window appears on the screen:

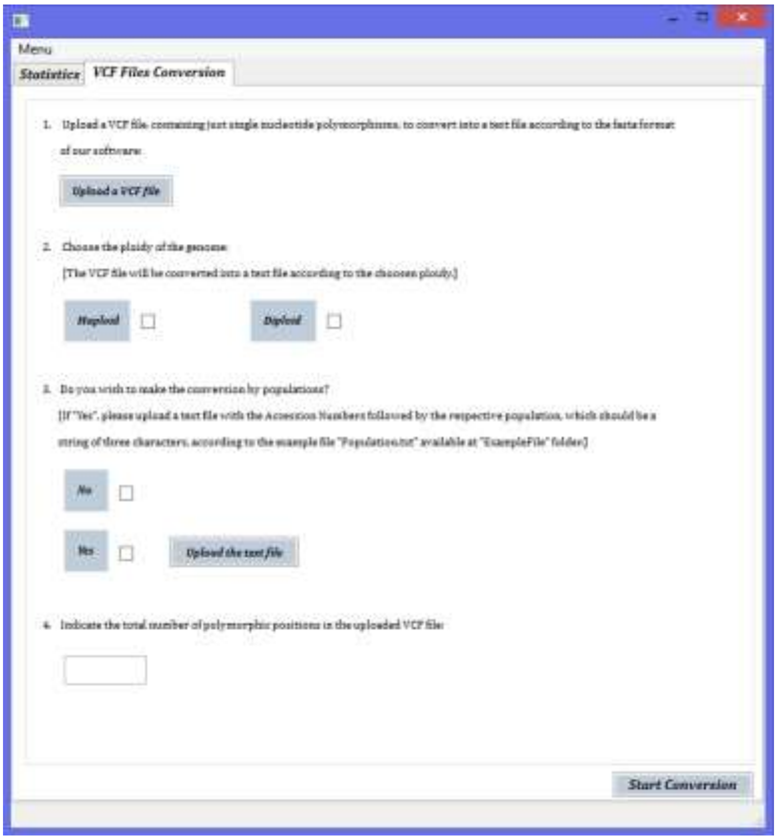

Figure 8. “VCF Files Conversion” tab from the GUI of DivStat software.

This tab is triggered when the user choose to upload a VCF file in the tab “Statistcs” to compute the statistics mentioned in the previous section. Nevertheless, it can be used whenever the user needs to convert a VCF file into a text file.

On the 2nd point of this tab, the user should identify the ploidy of the data, in order to enable a good reading and conversion of the VCF file into the text file.

On the 3rd point, the user should indicate whether the information about the population should be considered or not. In the affirmative case, the user should upload a text file with the information on the populations. More precisely, the file should contain the identification of the individual samples followed by the corresponding population indicated by a string of three characters, according to the following example:

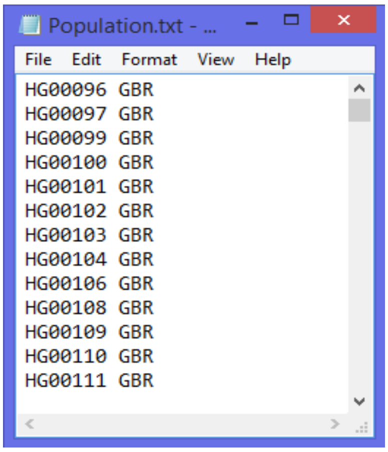

Figure 9. Example of an iutput text file with the information about the population (a string with three characters) of each genome (identified by its accession number).

Otherwise, all sequences are considered as belonging to the same population.

On the last point, the user should indicate the total number of polymorphic positions that are stored in the inputted file.

The output of this operation will be a text file with the data in the fasta format , which is similar to those shown in the figures 2. and 3. of the previous section (without and with missing data, respectively).

After file conversion, the user can proceed to the computation of the statistics.

2. *Command Line version – cmd:*

If the user needs to analyze more than one file, the cmd version is a most suitable option. In this case, the user should create a folder containing all text or VCF files to be analyzed, for instance “folder”, and then open the file “call.py” to indicate the parameters to perform the analysis of all files. The parameters to be defined are:

- *type_file*: the type/extension of the inputted files;
- *positions_VCFs*: the number of polymorphic positions in each inputted VCF file. This parameter is just required in case of define type_file as “.vcf”;
- *ploidy*: The ploidy of the data. The possibilities are “Haploid” and “Diploid”;
- *starts*: start position in the complete genome of the inputted haplotype sequences in each file;
- *stops*: end position in the complete genome of the inputted haplotype sequences in each file;
- *window*: window size ;
- *increment:* window increment;

- *window_def:* to state whether the window is defined by "number of base pairs" or "number of segregating sites";
- *statistics:* dictionary with required statistics, which are marked as "YES" (those marked as "" are not computed);
- *input_way*: path to the folder with the text or VCF files to be analyzed by the software;
- *general_name*: general name of the text or VCF files on the inputted folder. All files on the folder should have the same prefix, which should be followed by consecutive numbers, for example, "example_1.txt", "example_2.txt", "example_3.txt", etc. In this case, the general name is "example_";
- *output_way*: path to the folder where output files should be saved;
- *Pop*: "YES" to compute the statistics by populations and "No" otherwise;
- *general_name_pop_file*: general name of the text files on the inputted folder with the populations information. All files on the folder should have the same prefix, which should be followed by consecutive numbers, for example, "Population_1.txt", "Population _2.txt", "Population _3.txt", etc. In this case, the general name is "Population _". Note that, this parameter is just required in the case of defining the parameters type_file as ".vcf" and Pop as "YES";
- *MD*: "YES" if the files have missing data and "NO" otherwise;
- *symbol:* symbol used on the files for missing data (just needed when MD="YES");
- *num_runs*: number of FASTA files on the inputted folder that should be analyzed. The software runs one time for each file.

The python file "call.py" is similar to the following:

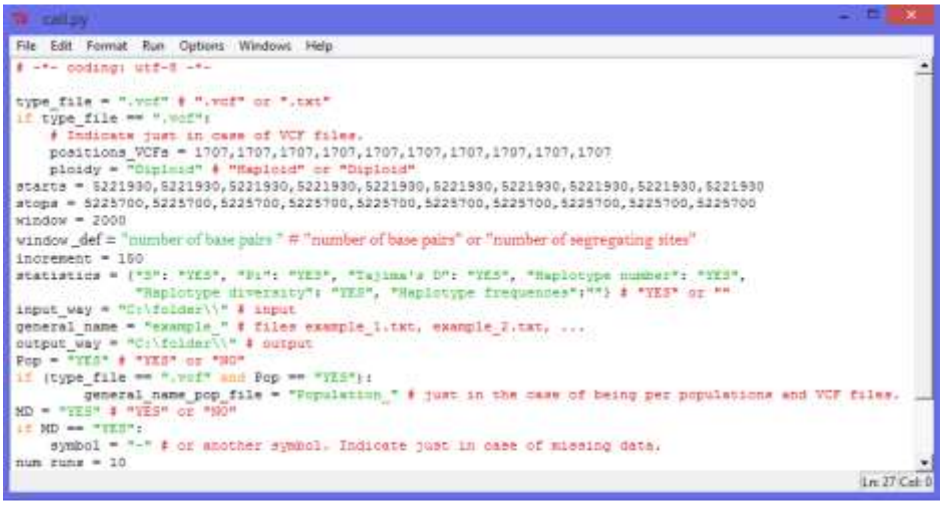

Figure 10. Python file named "call.py" in which the user should define the parameters to perform the analysis. Here, the user has VCF files with 1707

polymorphic positions and diploid genomes. The user defined the start and end positions of the inputted haplotype sequences of all files as being 5221930 and 5225700, respectively; the window size (defined by number of base pairs) and the window increment being 2000 and 150, respectively; the statistics required are S, Haplotype Number, Haplotype diversity, π and Tajima's D – window statistics; the files to be analyzed are in the folder named "folder" and each file has a name with prefix "example_"; the output should be in the same folder "folder"; the statistics should be computed per populations, being the information stored in files where the name has the prefix "Population_", and the files have missing data indicated by "-". Note that, the number of files on the inputted folder "folder" is 10, thus, DivStat should run 10 times with the defined parameters, one for each file in the folder.
